# Assessment of Quality of Life in Menopausal Periods: A Population Study in Kashan, Iran

**Published:** 2011-11-01

**Authors:** M Abedzadeh Kalarhoudi, M Taebi, Z Sadat, F Saberi

**Affiliations:** 1 Department of Midwifery, Kashan University of Medical Sciences, Kashan, Iran; 2 Department of Midwifery, Isfahan University of Medical Sciences, Isfahan, Iran

**Keywords:** Menopause, Quality of life, Physical activity, Income

## Abstract

**Background:**

Menopause is a physiological process in women's life. The aim of this study was to assess QOL and related factors among menopausal women in Kashan city in Iran.

**Methods:**

Seven hundred women aged 40–60 years were assessed using cluster sampling. Women with mental and physical problems or systemic diseases were excluded. Data were collected by standard questionnaire of quality of life in the menopause.

**Results:**

The mean menopausal age was 47.6±4.1 years. 55.6% of women had been postmenopausal for less than 5 years. The overall mean scores obtained for each domain was 2.82±1.64 for vasomotor, 2.71±1.2 for psychosocial, 2.46±0.99 for physical and 2.89±1.73 for sexual domains.

**Conclusion:**

Age, working status, physical activity, educational level, duration of menopause, income satisfaction, marriage satisfaction and the number of children in those living with family had influence on quality of life in menopausal women.

## Introduction

Menopause is a universal event in midlife, occurring around the age of 50 years in most developed countries.[1] Menopause is defined by 12 months of amenorrhea after the final menstrual period. It reflects complete, or near complete, ovarian follicular depletion and absence of ovarian estrogen secretion. Post menopause describes the period following the final menses; early post menopause is defined as the first five years after the final menstrual period. It is characterized by further and complete dampening of ovarian function and accelerated bone loss; and late postmenopause begins five years after the final menstrual period and ends with death.[2]

Headache, trouble sleeping, mood swings, vasomotor symptoms such as hot flash, and night sweats, somatic symptoms such as vaginal dryness, or atrophy and dysparonia, as well as psychological symptoms such as anxieties, difficulty in concentrating, overreacting to minor upsets, quickly being irritated, forgetfulness are symptoms of menopause and affect all dimensions of life quality.[3][4] The duration, severity, and impact of these symptoms vary from person to person, and population to population.[5] Some women have severe symptoms that profoundly affect their personal and social functioning, and quality of life (QOL).[6]

Quality of life (QOL) has been defined by the World Health Organization as the “individual’s perceptions of their position in life in the context of the cultural and value systems in which they live and in relation to their goals, expectations, standards and concerns”.[7] Quality of life is the main goal of health care and a significant factor for individual health and it is used to plan and evaluate health care programs.[8] Various validated tools have been used to determine the influence of the climacteric over QOL, among them the menopause-specific quality of life questionnaire (MENQOL) proposed by Hilditch et al.[9][10]

There are many researches which assessed QOL in menopause women. Some studies have shown no significant changes in QOL in menopausal women.[11][12][13][14] However, several indicated that QOL is impaired in menopausal women.[15][16][17][18] Many studies showed that some of demographic characteristics in postmenopausal women such as age, marital status, educational level, social and economical level, marriage satisfaction and the number of children who lived with the family were among other factors affecting the post-menopausal life.[19][20]

The age of menopause has not changed in past centuries; however the life expectancy has gradually increased. Considering the fact, life span is 84 years now,[21] today women spend one third of their life time after menopause.[22] It makes the postmenopausal as important as before menopausal life.[23] Postmenopausal women are one of most ignored groups and there are few research conducted on their quality of life[24] and results of these studies are controversial, therefore this study was performed to assess QOL and related factors among menopausal women in Kashan City, Central Iran.

## Materials and Methods

This was a cross-sectional study and the study population consisted of all menopausal women supported by the health care centers in Kashan City, Central Iran that is located in Isfahan Province. Menopause was defined by 12 months of amenorrhea after the final menstrual period.[2]

In this study, 700 women with natural menopause were selected using cluster sampling method. Women with mental and physical problems or systemic diseases were excluded. This study was conducted in 2008 after approval by Ethics Committee of Kashan University of Medical Sciences and prior to receipt of a consent form that was signed by the participants.

Data were collected by the latest edition (2004) of the standard questionnaire of quality of life in the menopause from the Women's Health Society of Toronto, Canada (MENQOL).[25] Content validity was used to assess the validity of questionnaire, and Cranach's alpha was used to assess the reliability. This questionnaire was a 29-item validated instrument that evaluated the effects of the items, divided into four domains, physical (16 items), vasomotor (3 items), psychosocial (7 items), and sexual (3 items) on quality of life in postmenopausal women.

The systematic scoring for each of the four MENQOL domains was identical. The seven-point Likert scale used during the administration of the MENQOL was transformed for scoring and data analysis. For each of the 29 items, this seven-point Likert scale ranged from 0 to 6. A “zero” was equivalent to a woman responding “no”, indicating that she had not experienced this symptom in the past month. Scores “one” through “six” indicated symptoms that the women experienced and increasing levels of bother experienced that corresponded to “1” through “6” check boxes on the MENQOL. Once each item has been manipulated into a 0–6 score, each domain was scored by averaging the manipulated values. Hence, the average for each domain was constrained between 0 (not at all a problem; respondent selected “no” for each item in the domain) and 6 (respondent reported experiencing each symptom in the domain at the highest degree of bother).

In these women, a number of demographic variables were recorded, including age, age of menopause, marital status, educational level, working status, the number of children living with them, marriage satisfaction and income satisfaction; they were also asked about lifestyle measures included smoking status and exercise.

Data were analysed using SPSS software (Version 15, Chicago, IL, USA) and presented as means, standard deviations, percentages, and confidence intervals. Differences in the MENQOL domain scores according to socio-demographic characteristics were compared by student t- test and analysis of variance (ANOVA). Multivariate analysis of variance was used to assess the influence of different variables on QoL; the four domains of QoL were considered as a dependent variable and socio-demographic variables as independent variables. The Colmogorov-Smirnov test was used to assess samples normality. A p value less than 0.05 was considered significant.

## Results

In this study 700 menopause woman aged 40-60 years were assessed. The Colmogorov-Smirnov test showed that samples had equal variances. The mean age of the women was 53.8±4.25 years and the mean menopausal age was 47.6±4.1 years. 55.6% of women had been menopause for less than 5 years. The mean number of children was 4.9±1.98; 1.1% of them had no children. Most of the women (51%) had elementary education; 98.3% were housewives; 89.9% had spouse, 98.3% had never smoked and 24.7% of the women reported physical exercise in their daily life. 73.6% of women had income satisfaction and 89% had marriage satisfaction (Table 1). MENQOL domains are shown in Table 2.

The scores in the four domains of the MENQOL questionnaire at different ages were also shown in Table 3. In physical, psychosocial and sexual domain scores, women aged 55–60 years reported higher quality of life scores than 40-44 and 45-50 and 50-54 year old respectively, but the differences were significant only for sexual domain (2.93±1.8; 2.93±1.62; 2.83±1.7; 3.04±1.45 respectively for 40-44 years, 45- 49 years; 50-54 years and 55-60 years ; p=0.04).

Spouse women had higher QOL score in vasomotor and psychosocial domains but differences were not statistically significant (vasomotor 2.83±1.62 vs. 2.70±1.71, p=0.55; psychosocial 2.72±1.19 vs. 2.65±1.14, p=0.64; physical 2.44±1.00 vs. 2.55±0.91; p=0.36).

Number of children living with their parents did not significantly impact on the vasomotor, psychosocial, and physical domains scores (P>0.05). However, in the sexual domain women who had not children had significantly lower scores from those who had children (2.59±1.82; 3.02±1.76; 2.89±1.70 respectively; p=0.007).

The menopause-specific quality of life improved with the level of education. Women with high school and university education had significantly lower scores compared with all the other educational levels, in psychosocial and physical domains (1.99±1.20, p<0.001; and 1.66±0.88, p=0.01; respectively).

Also, the effect of working status on QOL was evaluated. Housewives women had higher impaired QOL than employed women in all domains. But there was an only significant difference between two groups in physical and psychosocial domains (vasomotor 2.82±1.63 vs. 2.58±1.60; p=0.61; psychosocial 2.73±1.19 vs. 1.82±1.18; p=0.009; physical 2.46±0.99 vs. 1.87±1.07; p=0.04; sexual 2.90±1.76 vs. 2.36±2.24; p=0.31).

Women who had physical activity had lower QOL score in all domains and there were significantly differences in vasomotor, physical and psychosocial domains (vasomotor 2.50±1.63 vs. 2.92±1.62; P=0.003; psychosocial 2.33±1.19 vs. 2.83±1.16; P<0.001; physical 2.15±0.98 vs. 2.55±.97; P<0.001; sexual 2.68±1.65 vs. 2.97±1.80; P=0.07).

Women who smoked had higher QOL score in vasomotor, physical and psychosocial domains but differences were not significant (vasomotor 2.58±1.55 vs. 2.82±1.63, p=0.61; psychosocial 2.96±1.21 vs. 2.71±1.19, p=0.47; physical 2.40±1.14 vs. 2.45±.97, p=0.83; sexual 3.21±1.52 vs. 2.89±1.77; p=0.55). In the psychosocial, physical, and sexual domains, women who had menopausal duration less than 5 years ago had lower scores from those who had menopausal duration 5 or more years. But there was only significant difference between two groups in physical domain (vasomotor 2.83±1.67 vs. 2.80±1.60, p=0.80; psychosocial 2.65±1.22 vs. 2.77±1.17, p=0.20; physical 2.35±1.01 vs. 2.54±.97; p=0.01; sexual 2.76±1.70 vs. 3.01±1.81; p=0.07).

Income satisfaction was associated with psychosocial and sexual domains scores; women who had income satisfaction had significantly lower scores compared with other groups (vasomotor 2.81±1.64 vs. 2.82±1.62, p=0.97; psychosocial 2.61±1.17 vs. 3.00±1.18, p<0.001; physical 2.43±.99 vs. 2.51±1, p=0.33; sexual 2.76±1.74 vs. 3.28±1.79, p=0.001). Marriage satisfaction had association only with psychosocial domain of QOL (vasomotor 2.79±1.63 vs. 2.96±1.62, P=0.39; psychosocial 2.66±1.17 vs. 3.14±1.20, P=0.001; physical 2.43±0.98 vs. 2.65±1.02.90, P=0.07; sexual 2.89±1.75 vs. 2.90±1.929; P=0.97).

Multivariate analysis of variance showed that in vasomotor domain, exercise; in physical domain, education and exercise; in psychosocial domain, education; income satisfaction; exercise and marriage satisfaction and in sexual domain, income satisfaction were all predictors for better quality of life in menopausal women.

**Table 1:** Characteristics of menopausal women aged 40–60 years.

**Variables**	**No.(%) **
Age(years)	
40–44	15 (2.1)
45–49	82 (11.7)
50–54	279 (39.9)
55–59	324 (46.3)
Education level	
Illiterate	299 (42.7)
Elementary	333(47.6)
Guidance	24 (3.4)
High school and University	44 (6.3)
Working status	
Employee	12 (1.7)
Housewife	688 (98.3)
Marital status	
Spouse	629(89.9)
Without spouse	71 (10.1)
Menopausal Duration	
Less than 5 years ago	311 (44.4)
5 years or more	389 (55.6)
Number of children living with them	
0	168 (24)
1-2	360 (51.4)
≥3	172 (24.6)
Exercise	
Yes	173 (24.7)
No	527 (75.3)
Smoking	
Yes	12 (1.7)
No	688 (98.3)
Income Satisfaction	
Yes	515 (73.6)
No	185 (24.6)
Marriage Satisfaction	
Yes	623 (89)
No	77 (11)

**Table 2:** Mean of QOL scores in different domains.

**QOL Domains**	**Mean±SD**
Vasomotor	2.82±1.64
Psychosocial	2.71±1.2
Physical	2.46±0.99
Sexual	2.89±1.73

**Table 3:** Mean scores per domain in menopausal women according to socio-demographic characteristics.

**Characteristic **	**Vasomotor **	**Psychosocial **	**Physical **	**Sexual **
Age (years)				
40–44	1.73±0.45	2.37±1.28	2.17±1.2	2.93±1.8
45–49	1.43±0.15	2.71±1.08	2.37±1.01	2.93±1.62
50–54	2.88±1.7	2.67±1.22	2.43±1.03	2.83±1.7
55 –60	2.74±1.6	2.76±1.17	2.51± 0.94	3.04±1.45
*P*-value	*P*=0.48	*P*=0.29	*P*=0.12	*P*=0.04
Marital Status				
Spouse	2.83±1.62	2.72±1.19	2.44±1.00	2.90±1.76
Without Spouse	2.70±1.71	2.65±1.14	2.55± 0.91	-
*P* value	*P*=0.55	*P*=0.64	P=0.36	-
Number of Children living with them				
0	2.59±1.58	2.58±1.17	2.42±0.93	2.59±1.82
1 – 2	2.92±1.63	2.79±1.17	2.48±1.02	3.02±1.76
≥ 3	2.80±1.68	2.67±1.22	2.45±0.99	2.89±1.70
*P*-value	*P*=0.61	*P*=0.23	*P*=0.54	*P*=0.007
Education				
Illiterate	2.94±1.66	2.91±1.13	2.59±0.96	2.84±1.82
Elementary	2.78±1.63	2.64±1.19	2.44±0.98	3.02±1.70
Guidance	2.50±1.22	2.46±1.06	2.41±1.04	3.07±1.60
High School and University	2.40±1.62	1.99±1.20	1.66±0.88	2.27±1.87
*P*-value	*P*=0.16	*P*=0.000	*P*=0.01	*P*=0.84
Working status				
Housewife	2.82±1.63	2.73±1.19	2.46±0.99	2.90±1.76
Employee	2.58±1.60	1.82±1.18	1.87±1.07	2.36±2.24
*P* value , t, 95% CI	*P*=0.61	*P*= 0.009 t=2.629 (CI 0.23 to 1.59)	P=0.04 t=2.059 (CI .02 to 1.16)	*P*=0.31
Exercise				
Yes	2.50±1.63	2.33±1.19	2.15±0.98	2.68±1.65
No	2.92±1.62	2.83±1.16	2.55±0.97	2.97±1.80
*P* value , t, 95% CI	*P*= 0.003 t=-2.938 (CI -0.69 to -0.26)	*P*= 0.000 t=-4.914 (CI -0.70 to -.30)	*P*= 0.000 t=-4.661 (CI -0.56 to -0.23)	*P*=0.07
Smoking				
No	2.58±1.55	2.96±1.21	2.40±1.14	3.21±1.52
Yes	2.82±1.63	2.71±1.19	2.46±0.99	2.89±1.77
*P*-value	*P*=0.61	*P*=0.47	*P*=0.83	*P*=0.55
Menopausal Duration				
Less than 5 years	2.83±1.67	2.65±1.22	2.35±1.01	2.76±1.70
5 years and higher	2.80±1.60	2.77±1.17	2.54±0.97	3.01±1.81
*P*-value , t, 95% CI	*P*=0.80	*P*=0.20	*P*=0.01 t= 2.520 (CI -0.33 to -0.04)	*P*=0.07
Income Satisfaction				
Yes	2.81±1.64	2.61±1.17	2.43±0.99	2.76±1.74
No	2.82±1.62	3.00±1.18	2.51±1.00	3.28±1.79
*P* value , t, 95% CI	*P*=0.97	*P*= 0.000 t=-3.876 (CI -0.58 to -0.19)	*P*=0.33	*P*=0.001
Marriage satisfaction				
Yes	2.79±1.63	2.66±1.17	2.43±0.98	2.98±1.75
No	2.96±1.62	3.14±1.20	2.65±1.02	2.90±1.92
*P*-value , t, 95% CI	*P*=0.39	*P*=0.001 t=3.398 (CI 0.20 to 0.76)	*P*=0.07	*P*=0.97

## Discussion

The aim of this cross-sectional study was assessing QOL, using the MENQOL, among menopausal women and determining correlation between QOL and its determinants. Our findings showed that menopausal women had worse QOL in sexual and vasomotor domains. Several studies indicated that QOLwas impaired in menopausal women,[15][16][17][18] because menopausal period is related with several physical and mental changes that may impact women’s health outcomes.

Previous studies that evaluated the association between menopausal symptoms and socio-demographic and lifestyle factors, reported that lower socioeconomic status, education, length of menopause, physical activity, working status, and age were related to QOL,[5],[18],[26][27][28] the results were confirmed in this study too. In this study, number of children at home had a significant effect on sexual domain scores, which was not consistent with the results of a study from Iran,[29] but confirmed another study.[19] Blumel et al. believed that QOL decreased with excess of children. [15] Our results showed that marital status did not have any association with QOL. In a study, it was reported that there was a correlation between marital status and psychological and sexual QOL,[30] which had conflict with our results.

We found that physical and psychosocial quality of life scores in women with high school or university education were lower than women who had lower educational levels. In several studies, they concluded that women who had more than a high school educational level had experienced less disturbing and fewer symptoms during menopause.[18][27][31][32] In two studies, it was shown that educational level had a significant effect on all QOL domains.[18][28] However, in another study in which the overwhelming majority of the women had 12 or less years of education, it was demonstrated that those with a lower educational level had a low psychosocial score.[15] In general, a higher educational level was associated with better health, income and more opportunities in women's social and working life. The reason for the higher physical quality of life scores in menopausal women with higher educational levels may be due to their having more advantages in reaching the health services regularly, being informed and getting advice from physicians.

Working status had significant effect on physical and psychosocial domains. Also in two studies, researchers have found that being employed had a significant effect on physical and psychosocial QOL domains.[18][28] This is similar with our results. Blumel et al. also found that housewife women had higher score of QOL in all domains.[15] Kakkar found that working women seemed to suffer more from psychological symptoms whereas non-working women showed a greater incidence of somatic symptoms.[32] However, employed women had a strong social network connection and for this reason they did not have only higher self steam, but also had better social support and QOL.

Quality of life improved with performing exercise in the vasomotor, psychosocial and physical domains. This finding was compatible with results of Williams et al.’s study.[18] Also Lorenzi et al. found that exercise activity had association with higher QOL.[33] Exercise had beneficial effects on women’s mood, general well being, and sleep disorders and even on cognitive functions.[34] Women, who did not have exercise activity, had poor mental and physical health status.[35]

In this study, cigarette smoking was shown to have no significant effect on QOL domains. In Williams et al.’s study, the results showed that smoking status impacted significantly on psychosocial, physical, and vasomotor domains and never smokers had better scores than smokers.[18] In another study, the results showed that smoker women had lower QOL.[33] Cigarette smoking was shown to increase the risk of hot flashes,[5][33][36] and other physical and psychological symptoms experienced during menopause.[34] This conflict may be due to lower number of women smoker in our study.

In examining the quality of life scores according to length of menopause, menopausal duration less than 5 years had significant differences in physical domain. The results were confirmed by other studies. [27],[28] A study reported that women in early years of post menopause had lower QOL in vasomotor, psychosocial and physical domains.[16]

We found the physical and psychosocial quality of life scores in women who had income satisfaction to be lower than women in other group. This result was confirmed in several studies[13],[28],[37] but other studies have not shown association between income and QOL.[27],[29],[31] High income level may be increased by more access to health care centers and lead to a health promotion.

In our study, marriage satisfaction was shown to have a significant effect only in psychosocial domain of QOL. Results of several studies showed that marriage satisfaction had association with QOL especially in psychological health status[12],[19],[30] That is similar with our results. However comfort and happiness develops by marriage relations and losing or separating from husband and has a big impact on the psychological health of menopausal women.

Our study had some limitations. Women were asked to recall symptoms in the past four weeks. Although recall could be differentially biased based on different characteristics of women, we believe that this is a reasonable time frame for recall of many of our questions. Furthermore, since this is a cross-sectional study, we evaluated the association between factors and quality of life. We were unable to evaluate the impact of these factors on change in quality of life over time.

Despite these limitations, our study had several strengths. The information obtained for the MENQOL was self-reported and may have been affected by personality and social circumstances, yet self-report is an appropriate method to obtain information on perceptions, such as bothersome symptoms. In addition, the MENQOL questionnaire has been validated for use in postmenopausal women and has been used successfully by other studies too.[15][16][18][29][30]

The study results confirmed the impact of menopausal symptoms on health-related quality of life through the use of the MENQOL questionnaire. Results from this study showed that apart from symptoms that women experienced during menopause, socio-demographic factors also affected the quality of life of women during menopause.[38] In general, being older, being housewife, having less physical activity, lower education, duration of menopause more than 5 years, income and marriage un satisfaction and the number of children living with family more than 3 resulted in poorer menopause-specific QOL on many of the MENQOL domains.
